# Standardizing the Management of Tracheostomy Dislodgement in Patients With Subglottic Stenosis

**DOI:** 10.7759/cureus.91412

**Published:** 2025-09-01

**Authors:** Danielle A Hahn, Gisele J Wakim

**Affiliations:** 1 Anesthesiology, University of Miami Miller School of Medicine, Jackson Memorial Hospital, Miami, USA

**Keywords:** emergent airway, subglottic stenosis, tracheostoma care, tracheostomy decannulation (td), tracheostomy dislodgement

## Abstract

A 74-year-old female with tracheostomy due to laryngeal stenosis presented with a dislodged tube and significant stomal stenosis, making reinsertion challenging. Initial airway interventions included repeated failed reinsertion of tracheostomy tubes and unsuccessful endotracheal intubation. An emergent endotracheal tube was passed through the stoma by the anesthesia team after patient desaturation, and the patient was stabilized and transferred for specialized care. After successful intubation, the otolaryngologist performed tracheostomy revision. This case highlights airway management complexities in tracheostomal stenosis and underscores the need for a systematic approach and access to surgical specialists for successful intervention.

## Introduction

Tracheostomy is a surgical airway used in patients who are dependent on mechanical ventilation or require prolonged airway protection. The procedure has several potential complications, with one of the more dangerous including inadvertent tracheostomy tube decannulation, which may lead to loss of a secure airway or potentially rapid desaturation. Bhatia et al. [1] demonstrated that stomal stenosis may develop over time, often due to repeated tube changes, excess granulation tissue, or chronic inflammation or infection. Subglottic narrowing or stomal stenosis may make airway rescue particularly challenging, as it can prevent both successful oral intubation and tracheostomy reinsertion. Halum et al. [2] and Morris et al. [3] demonstrated that there is significant morbidity and mortality attached to tracheostomy decannulation, which only increases when reinsertion is complicated by stomal narrowing. Bontempo and Manning [4] found that after dislodgement of a tracheostomy tube, even if long-term, the airway can become non-patent within hours due to rapid stomal contraction. Current guidelines provide algorithms for management of accidental tracheostomy decannulation, but do not specifically address the added challenge of stomal stenosis. Prompt identification of stomal stenosis and preparation for potentially emergent airway challenges is essential, as delay in airway securement can be life-threatening. Despite its potentially fatal nature, there is limited literature describing the combined challenge of tracheostomy dislodgement in the setting of stomal stenosis. Increased awareness and understanding of this critical scenario are needed to inform both preventative strategies and acute management protocols. In this case, a 74-year-old patient with a long-term tracheostomy secondary to laryngeal stenosis presented with tracheostomy dislodgement, with reinsertion complicated by stomal stenosis.

## Case presentation

**Figure 1:**
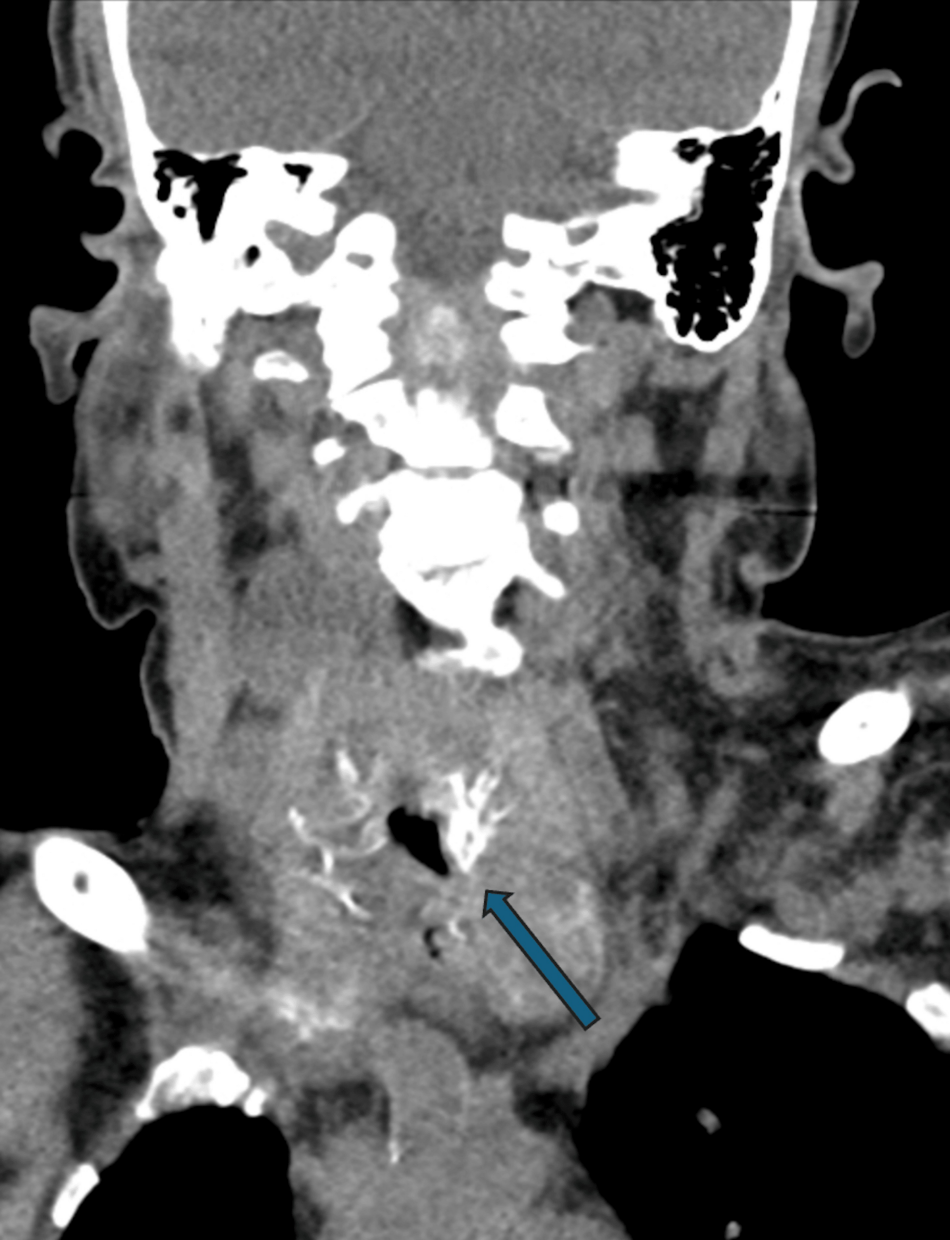
Coronal CT of the neck demonstrating a tracheostomy defect in the lower anterior mid-neck. Subglottic airway narrowing is present with associated soft tissue density surrounding the airway (blue arrow).

A 74-year-old female with tracheostomy for four years secondary to laryngeal stenosis, as well as altered mental status related to sepsis thought to be secondary to right upper thigh cellulitis, presented for evaluation of dislodged tracheostomy. As the patient was unable to give a history, it was unclear how long the tracheostomy had been displaced. The patient’s stoma had maintained some patency, but the degree of stomal stenosis did not permit reinsertion of tracheostomy at bedside in the intensive care unit (ICU). On examination, the patient was awake and alert, exhibited stridor but maintained her airway, saturating 100% on 2L nasal cannula, and remained hemodynamically normal. Attempts at tracheostomy reinsertion were made at bedside. Oxygen saturation transiently dropped to 88% during attempts at bedside reinsertion for approximately two to three minutes before supplemental oxygen restored it to 98%. A computed tomography (CT) scan of the neck revealed a tracheostomy tract defect in the lower anterior neck with associated subglottic airway narrowing and an adjacent soft tissue density, suggestive of a mass or enlarged thyroid gland (Figure 1). Around three hours after arrival, the patient was taken to the OR for an attempted awake tracheostomy insertion.

Anesthetic management of the patient during the attempted reinsertion procedure included the administration of ketamine and oxygen supplementation via nasal cannula. Ketamine was chosen to maintain spontaneous breathing while providing sedation. A fiberoptic scope was able to be passed through the small tracheostoma, but attempts made to advance a Shiley 6-0 cuffless tracheostomy (Medtronic, USA) were unsuccessful. In this case, fiberoptic guidance was used over initial direct surgical revision to limit trauma to the stenosed airway. A smaller, Shiley 4-0 cuffless tracheostomy was then advanced but was also unsuccessful. At this point, fiberoptic laryngoscopy was performed by anesthesia, the vocal cords were visualized, and the fiberoptic scope was successfully passed into the trachea. However, due to significant stenosis, an endotracheal tube (ETT) size 6 could not be advanced over the scope. After multiple attempts at securing the airway, the patient began to desaturate. The decision was made to emergently pass a size 4 ETT through the tracheostoma. The patient was initially bagged, but then initiated on mechanical ventilation. The patient was administered rescue breaths until she was saturating at 100%. Although the ETT through the stoma temporarily ensured ventilation, a definitive secure airway (e.g., a properly sized cuffed tracheostomy tube) could not yet be established. Upon removal of the ETT, the patient maintained adequate oxygenation with spontaneous respirations.

**Figure 2:**
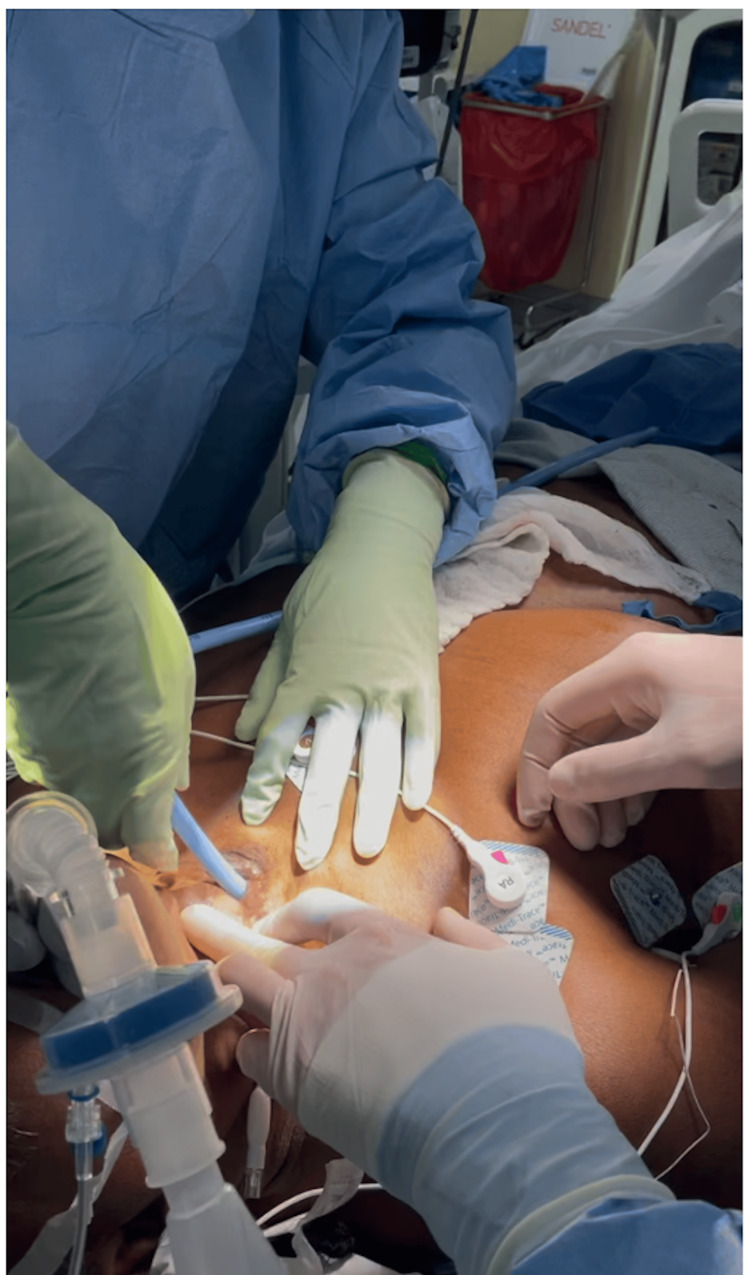
Image demonstrating tracheostomy dilation before tracheostomy tube reinsertion. Dilation is required to address the stenosis that was preventing reinsertion of the tracheostomy tube. Serial dilation was performed to allow reinsertion of a cuffed 6.0 tracheostomy tube in the presence of stomal stenosis.

**Figure 3:**
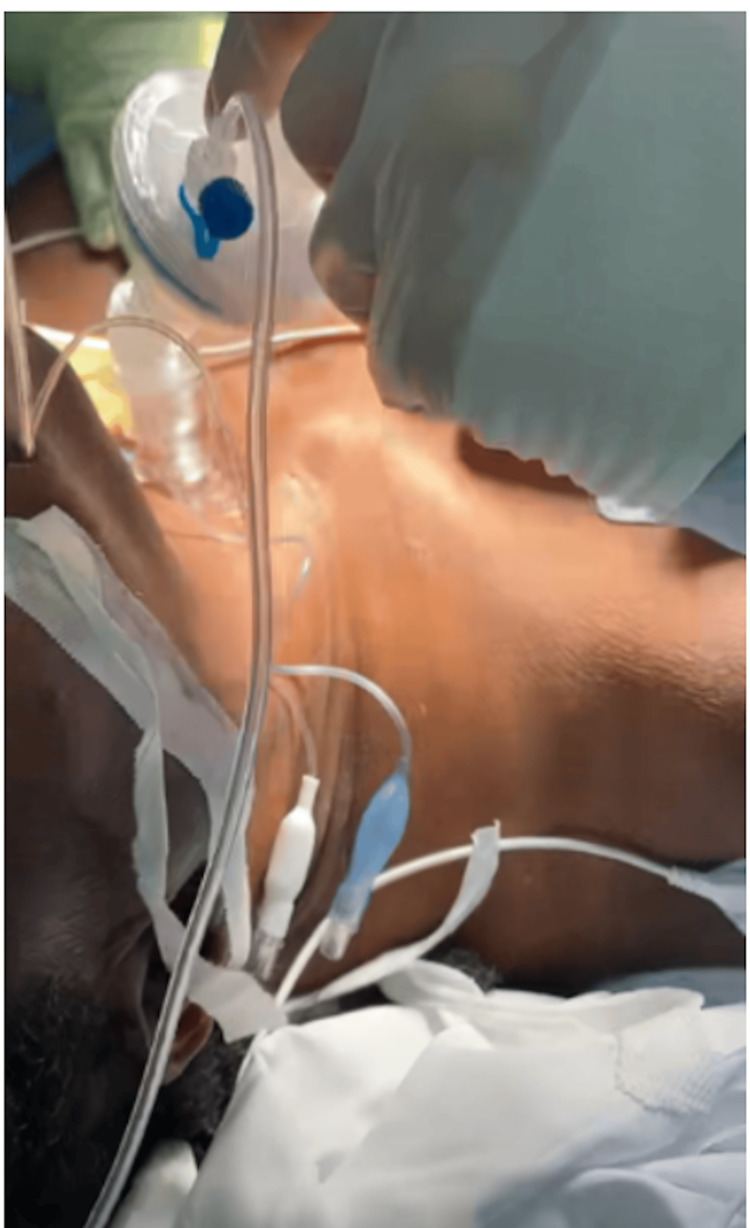
Image demonstrating tracheostomy tube reinsertion with size 6-0 cuffed endotracheal tube. Reinsertion was successful only after serial tracheal dilation.

Further attempts at dilation were aborted at this time due to the inability to establish a secure airway, and the general surgery team was unable to proceed with revision of the stenosed tracheostoma. At this juncture, it was recommended for the patient to be transferred to another facility for more specialized care by an otolaryngology team. The next morning, before the patient was transferred, she developed stridor and intercostal retractions, having trouble ventilating through her narrowed airway. At this point, the patient was emergently intubated with a size 4-0 ETT through the stoma, initially bagged with rescue breaths, and then initiated on mechanical ventilation. The decision was made to transfer the patient emergently for more specialized care. This time, the patient was advised to be seen not only by ENT, but also possibly by the cardiothoracic team in case a substernal tracheostomy placement was required. The Extracorporeal Membrane Oxygenation Team (ECMO) was on standby if the airway could not be maintained. The 4-0 ETT through the stoma was maintained to secure the airway for the transfer, and the patient was deemed hemodynamically stable for transfer by the ICU team. Upon arrival, the patient was brought to the OR in a supine position, and the airway was scoped, revealing a 4-0 ETT mainstemmed in the right lung. The tube was pulled out above the carina, and the airway was scoped from above, revealing a 50% subglottic stenosis with A-frame deformity and evidence of prior left posterior cordectomy. At this point, the decision was made to intubate from above and revise the tracheostomy only after securing the airway. ENT then proceeded with a Dedo laryngoscope for evaluation of the airway. A 6-0 cuffed tracheostomy was passed through the cords and through the subglottic stenosis with ease. The tube position was confirmed with appropriate end tidal CO_2_ and visualized symmetric chest rise. ENT then proceeded with tracheal dilation through the tracheostomy stoma through serial dilation up to 30 French using a bougie dilator (Figure 2). A 6-0 ETT balloon and Hegar dilators were used to dilate the stomal opening and surrounding skin. A 6-0 cuffed tracheostomy was placed in the airway with extreme resistance, and the tube was secured (Figure 3). Position of the tube was confirmed with direct visualization via a fiberoptic scope. Minimal bleeding occurred following insertion, and ventilation was unobstructed. The patient achieved good tidal volumes and was saturating 100% at this time. No hypoxia, aspiration, or cardiac events occurred during this process.

The patient's tracheostomy tube was secured, and the patient was transferred to the ICU for respiratory monitoring and complex patient care. The patient remained on mechanical ventilation and was initiated on propofol for sedation and to maintain a relaxed airway in the ICU setting. Three days later, the patient was eventually able to be weaned off sedation and ventilation and discharged with a secure tracheostomy tube in place.

## Discussion

**Figure 4:**
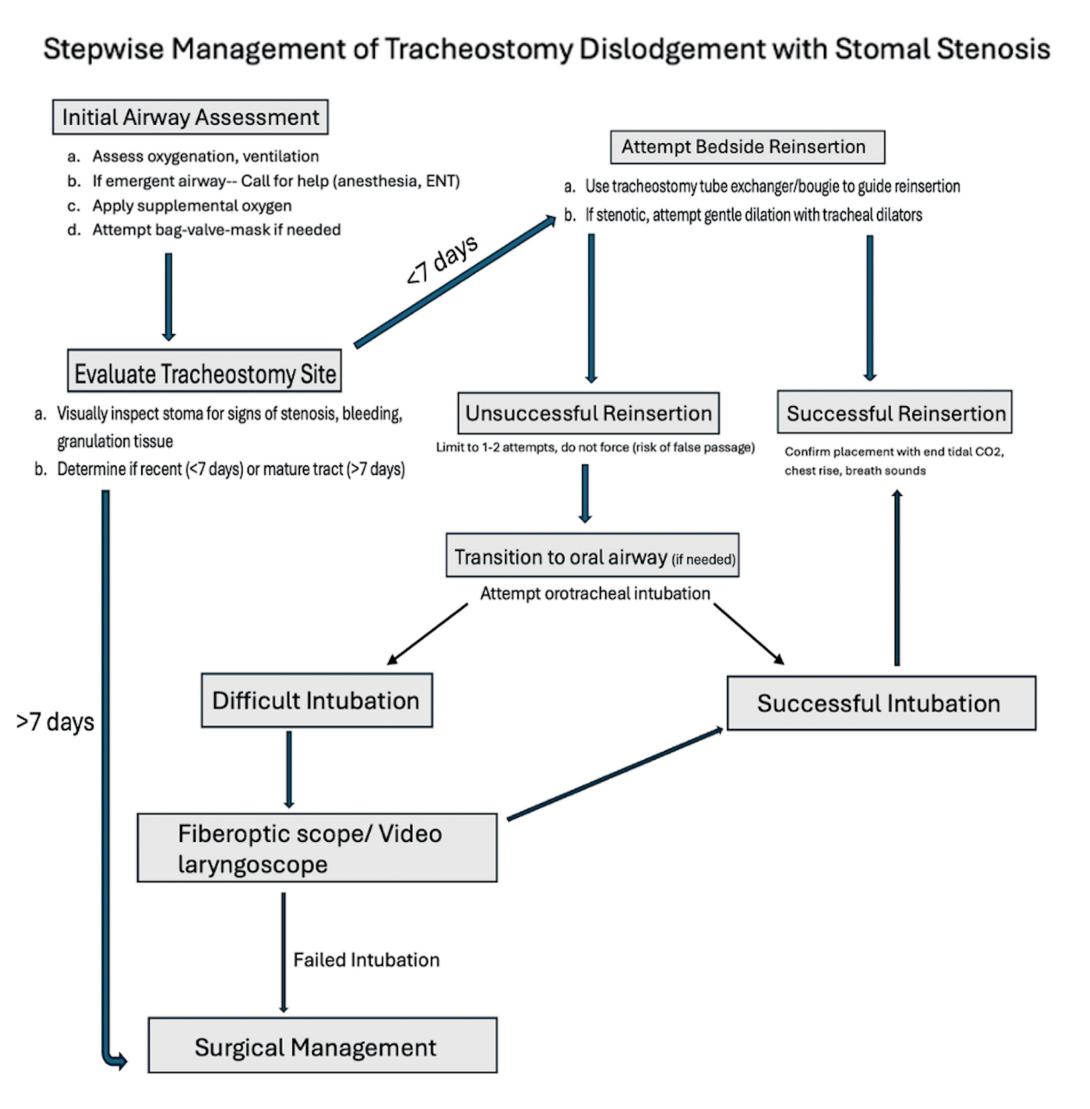
Chart demonstrating the algorithm for managing an airway after tracheostomy dislodgement with subglottic stenosis. Image credit: Danielle Hahn, adapted from the National Tracheostomy Safety Project (NTSP) guidelines.

Tracheostomy tube dislodgement is a common, life-threatening tracheostomy complication, and stomal stenosis only further complicates the airway management in these patients. McGrath et al. [5] illustrated that early recognition and rapid response strategies for reinsertion are critical to prevent potential airway crises and life-threatening scenarios. From the surgical perspective, there are strategies that can be implemented during the insertion of a tracheostomy to reduce the risk of postoperative dislodgement, including proper tube selection and positioning. Current data suggest that surgeons should take measures to ensure that the initial tracheostomy tube is appropriately sized for the patient’s anatomy, with some tracheostomy patients with larger neck circumference or deep-set trachea requiring a longer tube to minimize the risk of decannulation. Bontempo and Manning [4] suggested that surgeons should assess for signs of tracheostomy tube malposition while the patient is still in the procedure room with monitoring of tracheostomy tube cuff pressures. The cuff should achieve a seal at low inflation pressures; high cuff pressures required to maintain a seal should raise suspicion for tube malposition. In such cases, the tube should be exchanged in the procedure room rather than risking dislodgement postoperatively. There are conflicting opinions on the use of sutures to secure the tracheostomy tube while the tract is still forming in the first 7-10 days post tube insertion. Halum et al. [2] and Jung et al. [6] agreed that current data do not show a significant reduction in the risk of premature tracheostomy dislodgement; however, many still choose to use sutures to help stabilize the surgical site and theoretically prevent postoperative bleeding. In this particular case, the patient did not have any factors that would indicate the need for a longer tube, and the initial operative notes were unavailable to identify any factors that would increase the risk of dislodgement. Despite the absence of identifiable factors that would increase the risk of dislodgement, this complication is likely never completely preventable. Current data suggest that preventative strategies may include regular stomal assessments on any tracheostomy patient presentations, as well as timely surgical evaluation if the physical exam shows evidence of tract narrowing. Like Jung et al. [6], McGrath et al. [5] agreed that patients with known high-risk features for narrowing of the tracheocutaneous tract, including a history of head and neck surgery or radiation therapy or prolonged tracheostomy dependence, should be more thoroughly examined and may benefit from routine ENT examinations to assess for stomal patency. Extreme care should be taken by anyone working at the bedside to prevent accidental decannulation in view of the delicacy of the tracheotomy tubes. This case also highlights the need for a stepwise approach for difficult tracheostomy reinsertion, as shown below. When standard reinsertion at bedside fails, alternative strategies should be implemented, as per Knight et al. (Figure 4) [7].

There should always be a facility-guided difficult airway plan for all emergent airways with various levels of responses depending on the level of complexity of the facility and the resources available on site, as per Knight et al. [7]. The purpose of the plan would be to stay organized during respiratory crises with well-defined roles for personnel, as well as to have allocated equipment and education on the available resources for each crisis to maximize patient safety. In the case of an acute airway crisis secondary to tracheostomy dislodgement with stomal stenosis, particularly if the dislodgement occurs outside the ICU or OR, as in this patient, the lack of immediate access to airway equipment or trained personnel may delay definitive airway management. This puts the patient at risk for stomal narrowing or closure. This case emphasizes the need for more current, well-defined protocols around emergency tracheostomy management. Morris et al. [3] and Jung et al. [6] found that many institutions currently recommend standardized tracheostomy emergency kits at the bedside of hospitalized tracheostomy patients, as well as in the emergency departments and ICUs. Table 1 demonstrates the contents of these emergency kits.

**Table 1:** Table demonstrating suggested contents of tracheostomy emergency airway kits to allow for urgent bedside intervention. Table credit: Danielle Hahn

Emergency Kit Contents	Function
Tracheostomy tubes (various sizes)	Eases bedside tracheostomy reinsertion
Suction	Removes excess secretions, increases visibility for potential intervention
Flexible suction catheter, red rubber catheter	Reinsertion guidance
Bag mask ventilation equipment	Ventilate patient
Oral intubation equipment	Secure the airway

## Conclusions

In this case, the patient was brought to the hospital from her residential nursing home, and it was unclear how long the tracheostomy had been dislodged. Even in a well-equipped facility, limited access to specialized ENT care for a complex respiratory patient resulted in a delay in securing the airway. While ICU teams, anesthesiologists, and otolaryngologists ultimately worked together to manage the complex airway post-patient transfer, some measures could have been taken to mitigate patient risk. This case highlights the need for a structured, multidisciplinary approach to tracheostomy dislodgement, particularly in patients with subglottic stenosis. While we proposed an airway management algorithm and emergency kit as potential tools, further validation in larger clinical settings is required. This case also raises awareness for the need for clear emergency protocols and provider training to prevent critical delays in management in the event of tracheostomy decannulation.
